# Exploring the heterogeneity of human exposure to malaria vectors in an urban setting, Bouaké, Côte d’Ivoire, using an immuno-epidemiological biomarker

**DOI:** 10.1186/s12936-019-2696-z

**Published:** 2019-03-11

**Authors:** Dipomin F. Traoré, André B. Sagna, Akré M. Adja, Dounin D. Zoh, Kouassi A. Adou, Kouassi N. Lingué, Issa Coulibaly, N’Cho Bertin Tchiekoi, Serge B. Assi, Anne Poinsignon, Mamadou Dagnogo, Franck Remoue

**Affiliations:** 1 Institut Pierre Richet (IPR), Institut National de la Santé Publique (INSP), Bouaké, Côte d’Ivoire; 2 MIVEGEC, Univ. Montpellier, CNRS, IRD, Montpellier, France; 3 Unité de Formation et de Recherche en Sciences de la nature (UFR SN), Université Nangui Abrogoua, Abidjan, Côte d’Ivoire; 4 Unité de Formation et de Recherche en Biosciences (UFR Biosciences), Université Félix Houphouët Boigny, Abidjan, Côte d’Ivoire; 5 Institut de Géographie Tropicale (IGT), Université Félix Houphouët Boigny, Abidjan, Côte d’Ivoire

**Keywords:** Urban malaria, salivary biomarker of exposure, *Anopheles*, Transmission risk

## Abstract

**Background:**

In some African cities, urban malaria is a threat to the health and welfare of city dwellers. To improve the control of the disease, it is critical to identify neighbourhoods where the risk of malaria transmission is the highest. This study aims to evaluate the heterogeneity of malaria transmission risk in one city (Bouaké) in a West African country (Côte d’Ivoire) that presents several levels of urbanization.

**Methods:**

Two cross-sectional studies were conducted in three neighbourhoods (Dar-es-Salam, Kennedy and N’gattakro) in Bouaké during both the rainy and dry seasons. Data on insecticide-treated net (ITN) use and blood samples were collected from children aged between 6 months and 15 years to determine the parasite density and the prevalence of *Plasmodium falciparum* and the level of IgG against the *Anopheles* gSG6-P1 salivary peptide, used as the biomarker of *Anopheles* bite exposure.

**Results:**

The specific IgG levels to the gSG6-P1 salivary peptide in the rainy season were significantly higher compared to the dry season in all neighbourhoods studied (all *p* < 0.001). Interestingly, these specific IgG levels did not differ between neighbourhoods during the rainy season, whereas significant differences in IgG level were observed in the dry season (*p* = 0.034). ITN use could be a major factor of variation in the specific IgG level. Nevertheless, no difference in specific IgG levels to the gSG6-P1 salivary peptide was observed between children who declared “always” *versus* “never” sleeping under an ITN in each neighbourhood. In addition, the prevalence of *P. falciparum* in the whole population and immune responders was significantly different between neighbourhoods in each season (*p* < 0.0001).

**Conclusion:**

This study highlights the high risk of malaria exposure in African urban settings and the high heterogeneity of child exposure to the *Anopheles* vector between neighbourhoods in the same city. The *Anopheles* gSG6-P1 salivary peptide could be a suitable biomarker to accurately and quantitatively assess the risk of malaria transmission in urban areas.

**Electronic supplementary material:**

The online version of this article (10.1186/s12936-019-2696-z) contains supplementary material, which is available to authorized users.

## Background

The fast urbanization of large African cities, the anarchic occupation of urban space and various socioeconomic conditions have major implications in the epidemiology of urban malaria. The presence of shallow waters, rice cultivation and gardening in an urban environment could lead to variation in malaria transmission [1]. In Bouaké, the second largest city of Côte d’Ivoire, several shallows have been transformed for rice farming and vegetable farming. This factor increases mosquito proliferation, in particular *Anopheles gambiae*, the major malaria vector in Africa, which is adapted to this urban environment and ensures a continuous transmission of malaria in several of the city’s neighbourhoods [2]. The presence of *An. gambiae* mosquitoes depends on local conditions, which explain the considerable variability of malaria distribution. Malaria transmission may vary from one region, neighbourhood and household to another, reflecting the concept of transmission hot spots [1, 3–5]. Even though malaria transmission in urban settings is generally considered low compared to rural areas, city dwellers could be considered at high risk of severe malaria because of their low acquired immunity specific to malaria, highlighting the particular health problem of urban malaria [6, 7].

The evaluation of malaria transmission is currently based on entomological methods (human-landing catch) and on parasitological assessments in human populations. However, these methods are labour-intensive and difficult to sustain on a large scale, especially when transmission and exposure levels are low (dry season, high altitude, urban settings or after vector control) [8, 9]. The entomological methods commonly used to assess human exposure to mosquito bites do not provide a measure of the individual exposure in a given area. In addition, these methods inevitably increase the hazard of the participants’ exposure to mosquito-borne infections and, therefore, cannot be used in children [10, 11].

To improve the evaluation of malaria transmission/exposure according to the World Health Organization (WHO) recommendations, much effort is being made to develop new indicators and methods at the individual level. Over the past few decades, several studies have shown that the measurement in human populations of antibody (Ab) responses to saliva molecules of vector insect was an adequate method to assess the human exposure level to vector bites and the risk of vector-borne disease [12, 13]. Specifically, the gSG6-P1 peptide (*An. gambiae* Salivary Gland Protein-6 peptide 1) of *Anopheles* saliva has been identified as a pertinent biomarker of *Anopheles* bites [14]. This salivary peptide is specific to the *Anopheles* genus, antigenic, easy to synthesize and highly conserved between *Anopheles* mosquitoes [14]. In particular, the human IgG response to the gSG6-P1 peptide was especially relevant as a biomarker in a context of low exposure to *Anopheles* bites, for example in urban settings and during the dry season [15, 16]. In one study, carried out in northern Senegal in 2013, this salivary biomarker was used to observe a considerable heterogeneity of human exposure to *Anopheles* between neighbouring villages in a low-transmission setting [16]. In addition, this biomarker was recently used to identify hotspots of malaria transmission in severe areas in Thailand [17]. It could therefore be applied to malaria surveillance and control (i) by assessing the level of heterogeneity of human exposure to *Anopheles* bites [18] and (ii) by evaluating the efficacy (Phase 3 study) and the effectiveness (operational level, post-implementation) of vector control strategies [19, 20]. Indeed, in low urban transmission areas in Dakar, Senegal, it was shown that human IgG responses to the gSG6-P1 peptide could be, at both the population and individual levels, a credible new alternative tool to assess the heterogeneity of exposure levels to *Anopheles* bites and malaria risk [15]. Moreover, by proving the usefulness of this biomarker for assessing the effectiveness of anti-malaria vector control in populations, it was shown that this biomarker could be used as a potential alternative to the standard entomological methods, especially in low-endemic areas and urban settings [20]. Previous results in the same areas, using this immunological biomarker, indicated that human exposure to *Anopheles* bites remained similar in both urban and rural areas, whatever the season [21]. Surprisingly, urban populations could therefore be as highly exposed to *Anopheles* bites as populations living in rural areas.

The major aim of the present study was, therefore, to explore the heterogeneity of human exposure to *Anopheles* bites in different neighbourhoods in Bouaké, Côte d’Ivoire, using the *An. gambiae* salivary biomarker (gSG6-P1). Additionally, in this context of urban exposure to the *Anopheles* vector, the potential impact of the declared use of insecticide-treated nets (ITNs) was evaluated on human–vector contact.

## Methods

### Study area

**Fig. 1:**
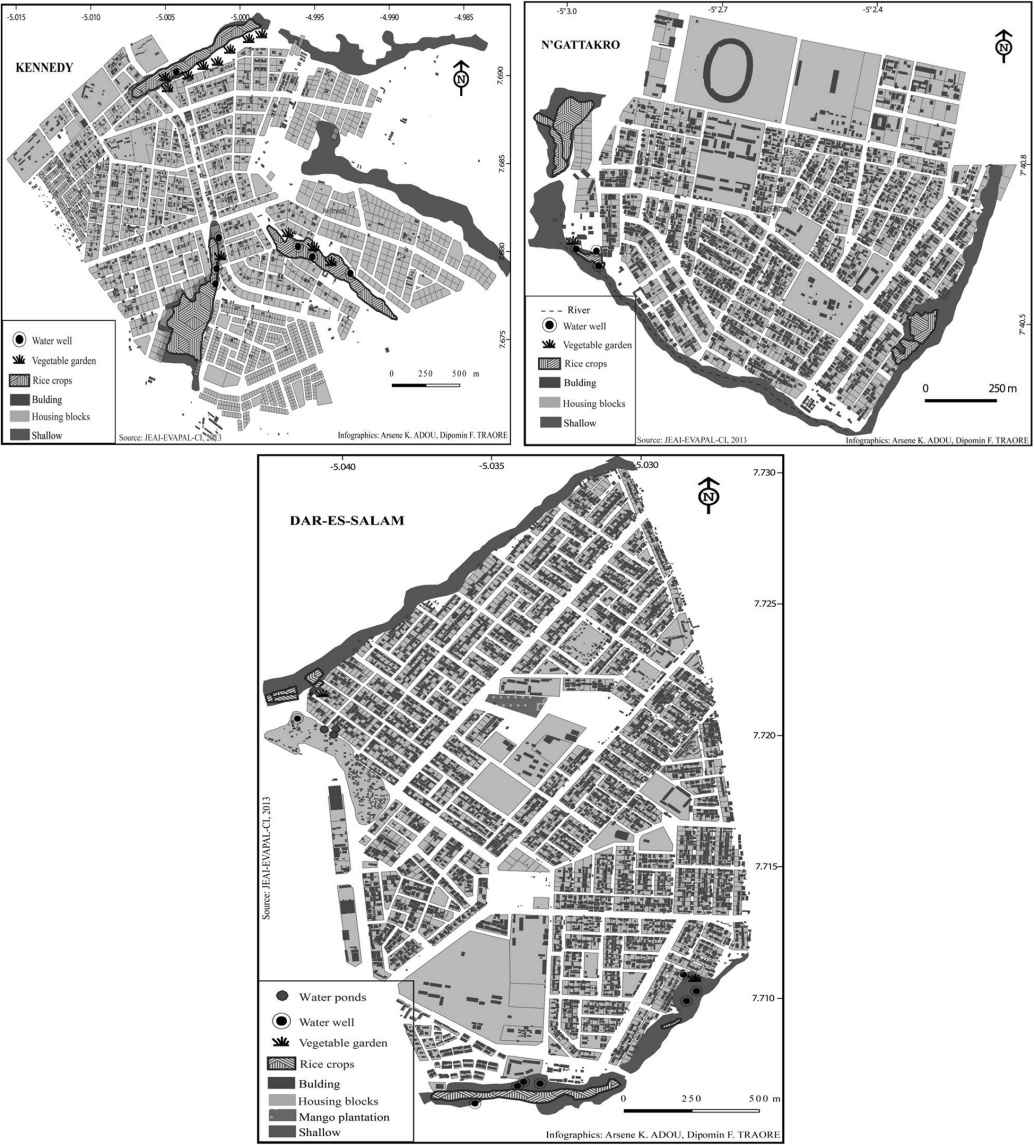
Characterization of Bouaké neighbourhood environment. This figure shows maps of different bio-ecological aspects of Bouaké neighbourhoods (**a** N’gattakro; **b** Kennedy; **c** Dar-es-salam), which could be potential breeding sites of *Anopheles*. People have transformed shallows to grow rice and vegetable crops and use well water for their field activities

In Côte d’Ivoire, malaria transmission is stable throughout the country and presents peaks during the rainy season [22]. This study was conducted in three neighbourhoods of Bouaké: Kennedy (KEN), N’gattakro (NGA) and Dar-es-Salam (DAR). The population of Bouaké is estimated at 536,719 inhabitants: 5000 inhabitants in KEN, 14,684 in NGA and 54,992 in DAR [23]. The Bouaké region is located in a climatic transition area that shows two seasons: the dry season and the rainy season. The annual average rainfall varies between 1000 and 1200 mm [24]. The urban area is crossed by many small watercourses located 500–800 m from each other. Apart from the city centre, all the Bouaké neighbourhoods studied are crossed by humid shallows (Fig. 1). For some years, most of these shallows have been used for gardening and as paddy fields. The neighbourhoods of NGA and DAR are both known for their paddy field areas and vegetable gardens. In contrast, in KEN, both types of urban farming are found in the centre of the neighbourhood.

### Population studied

Blood samples and a sociological questionnaire concerning epidemiological data and ITN use were collected during two periods: (1) in the rainy season (August 2014) and (2) in the dry season (April 2015). Households and children (6 months to 15 years) were randomly selected by the project’s sociological team. At each survey, children with an axillary temperature of 37.5 °C or higher or a recent history of fever (within 24 h of the survey) with a positive malaria rapid diagnostic test (RDT) were treated with anti-malarial drugs, as recommended by National Malaria Control Programme (NMCP). A total of 801 thick blood smear samples were collected from the children studied for parasitological measurements. Parasite density (parasitaemia) was calculated as the number of *P. falciparum* parasites per microliter of blood (PD = Number of trophozoites counted × 8000/Number of leukocytes counted); the geometric mean of parasitaemia was also calculated. In addition, capillary blood samples were collected in Microvettes^®^ microtubes (Sarstedt^®^, Marnay, France) and centrifuged to obtain serums, which were stored at − 20 °C for immunological study.

### Salivary peptide gSG6-P1

The gSG6-P1 peptide was designed using bioinformatics to maximize its *Anopheles* specificity and its immunogenicity, as previously described [14]. It was synthesized and purified (> 95%) by Genepep SA (Saint Jean de Védas, France). The peptide was shipped in lyophilized form and then resuspended in 0.22-µm ultra-filtered water and stored at − 20 °C for later use.

### Evaluation of human IgG antibody level

ELISAs (Enzyme-Linked Immunosorbent Assay) were carried out on individual sera to measure the IgG level to the gSG6-P1 peptide as previously described [16, 21]. Briefly, Maxisorp plates (Nunc, Roskilde, Denmark) were coated with gSG6-P1 (20 μg/ml) in PBS (phosphate buffered saline). After washing (demineralized water + Tween 0.1%), each serum was incubated in duplicate at 4 °C overnight at a 1/320 dilution (in PBS with 1% Tween 20). A biotinylated mouse anti-human IgG (BD Pharmingen, San Diego, CA, USA) was incubated at a 1/4000 dilution in PBS with 1% Tween (1.5 h at 37 °C) and peroxidase conjugated ExtrAvidin (Sigam, St. Louis, MO, USA) was then added (1/20,000; 1 h at 37 °C). Colorimetric analysis was carried out using ABTS (2.2-azino-bis (3 ethylbenzthiazoline 6-sulfonic acid) diammonium; Sigma) in 50 mM citrate buffer (Sigma, pH = 4, containing 0.003% H_2_O_2_) and the absorbance (OD) was measured at 405 nm. Individual results were expressed as: ΔOD = ODx − ODn, where ODx represents the mean of the individual optical density (OD) value in both wells with gSG6-P1 antigen and ODn the individual OD value in a blank well containing no gSG6-P1 antigen.

The positivity threshold (PT) of the IgG level to anti-gSG6-P1 was calculated using the following formula: PT = mean (ΔODneg) + 3SD. The ΔODneg mean of non-*Anopheles*-exposed individuals from Bordeaux (southwestern France) was zero. Consequently, PT = 0 and an exposed individual was then classified as an immune responder if the ΔOD was greater than zero.

### Statistical analysis

Data analysis was carried out using Graph Pad Prism^®^ (Graph Pad Software, San Diego, CA, USA). Values in each group did not assume a Gaussian distribution. The nonparametric Mann–Whitney U test was then used for the comparison of IgG levels in children between age groups and also between individuals who always slept under an ITN and those who never slept under an ITN. The nonparametric Kruskal–Wallis test was used to compare the three neighbourhoods. The Dunn post-test was used for multiple comparisons between neighbourhoods. All differences were considered as significant at *p* < 0.05. Excel software was used to calculate the mean geometrical parasitaemia of *P. falciparum*. The Chi square test was used to compare *P. falciparum* prevalence between neighbourhoods in both seasons.

### Ethics statement

This study followed the ethics principles recommended by the Edinburgh revision of the Helsinki Declaration. The present study was approved by the Ethics Committee of the Côte d’Ivoire Ministry of Health (June 2014; No. 41/MSLS/CNER-dkn). Written informed consent of all the parents or guardians of children who participated in the study was obtained before inclusion.

## Results

### IgG level against gSG6-P1 salivary peptide according to neighbourhoods

**Fig. 2:**
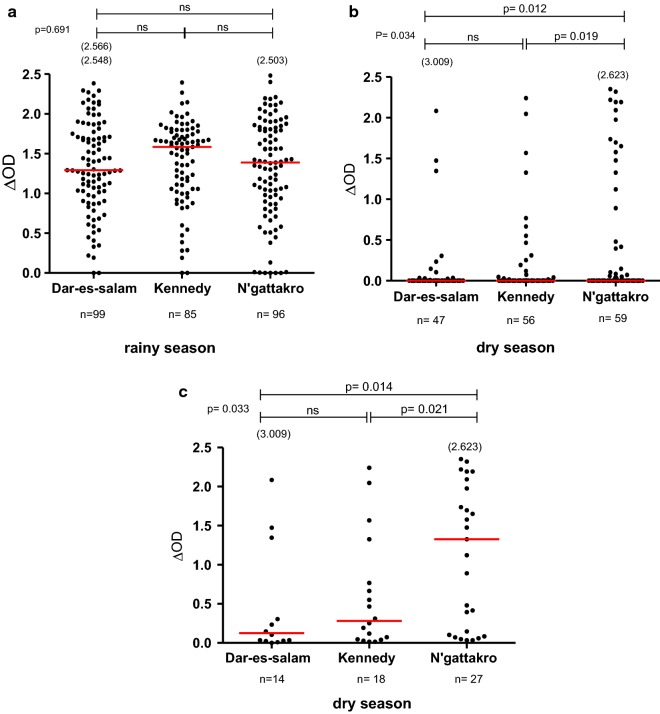
Comparison of IgG level with gSG6-P1 salivary peptide between neighbourhoods. Dot plots show the individual specific IgG level (∆OD value) to gSG6-P1 in the children of the neighbourhoods studied between the seasons: in the rainy season (*n* = 281; **a**) and the dry season (*n* = 161; **b**) for the whole “immune responders and not responders to the peptide gSG6-P1” population and for the only immune responders to gSG6-P1 (*n* = 59; **c**). Bars indicate the median value in each group. The number in parentheses on the figure above the dot plots indicates values above ∆OD = 2.5. The statistical results using the nonparametric Kruskal–Wallis test are indicated between the groups studied

To explore if different bio-ecological environments between neighbourhoods would influence individual exposure to *Anopheles* bites (Fig. 1), the specific IgG response in children was compared between the three neighbourhoods studied during the rainy and dry seasons, classically known for the periods of high and low exposure to *Anopheles* vectors, respectively. The first analysis showed that the level of specific IgG varied according to seasons and was higher in the rainy season compared to the dry season in all neighbourhoods (all *p* < 0.0001; Mann–Whitney test, data not shown). The second analysis compared the levels in specific IgG responses of children between neighbourhoods in the rainy season (Fig. 2a) and the dry season (Fig. 2b). In the rainy season, high specific IgG responses were observed in all the three neighbourhoods studied and no statistically significant difference was observed between them (*p* = 0.691; Kruskal–Wallis test). In contrast, specific IgG levels in the dry season differed between the three neighbourhoods (*p* = 0.034; Kruskal–Wallis test) despite the very low level of IgG medians. Specific IgG levels were significantly higher in NGA compared to KEN and DAR (*p* = 0.019 and *p* = 0.012, respectively). No significant difference of specific IgG level was observed between KEN and DAR. When taking into account the whole population studied in the dry season (responders and non-responders to the gSG6-P1 peptide), the median levels of specific IgG responses, even if very close to zero, remained different between neighbourhoods. Nevertheless, as it appeared to be difficult to differentiate the specific IgG medians at the population level between the different neighbourhoods, a second analysis was then set up considering only the IgG responder individuals to gSG6-P1 (i.e., IgG immune responders; Fig. 2c). A high median IgG level was observed in NGA, showing high individual values for some children, whereas very low IgG responses were detected in DAR and KEN. The specific IgG level remained significantly higher in NGA compared to KEN and DAR (*p* = 0.021 and *P* = 0.014, respectively) in the subpopulation of IgG immune responders.

### IgG levels against gSG6-P1 peptide in children according to age group

To evaluate the potential impact of children’s age on the specific IgG level between neighbourhoods, three age groups were first defined in both the rainy and dry seasons: 0–5, 6–10 and 11–15 years old. No significant difference in specific IgG level according to the three neighbourhoods was observed in the two seasons. However, there were few children aged from 11 to 15 years (n = 3; 14; 16 for DAR, KEN, NGA respectively, in the dry season) and, for this reason, they were grouped into one age group (6–15 years). Two age groups were then defined for immunological analysis: ≤ 5 and 6–15 years old (Additional file 1). The age-dependent IgG levels were then compared between the neighbourhoods studied in the dry and rainy seasons. The anti-gSG6-P1 IgG levels in children under 5 years old were higher in NGA than in DAR and KEN. This difference was only significant in the dry season (*p* = 0.0145). No significant difference was observed in children over 5 years old (6–15 years) between neighbourhoods in the two seasons. These age-dependent IgG levels were also compared within neighbourhoods in the dry and rainy seasons. No significant difference was observed (Additional file 1).

### Parasitological data

The prevalence of *P. falciparum* and the geometric mean of parasitaemia were calculated in the whole population and in individuals who were positive in specific IgGs to the gSG6-P1 peptide (i.e., immune responders) during both seasons (Table 1). In the whole population and in immune responders, the prevalence differed between neighbourhoods for each season (*p* < 0.0001, Chi square test) (Table 1). The prevalence was higher in KEN (90.58%; 90.36% in the whole population and immune responders, respectively) and NGA (92.71%; 91.40%) compared to DAR (50.56%; 56.70%) in the rainy season. Similar results were obtained in the dry season (Table 1). Moreover, the prevalence of *P. falciparum* did not vary between the two seasons in each neighbourhood. Similar trends between neighbourhoods and seasons were observed for the geometric mean of *P. falciparum* parasitaemia (Table 1).

**Table 1:** Characterization of prevalence and parasite density of *Plasmodium falciparum* average according to neighbourhood during the rainy season and dry season

Neighbourhood	Rainy season			p-value	Dry season			p-value
	Dar-es-salam	Kennedy	N’gattakro		Dar-es-salam	Kennedy	N’gattakro	
Total population								
Prevalence	56/99 (56,56%)	77/85 (90,58%)	89/96 (92,71%)	***	24/47 (51%)	53/56 (94,64%)	52/59 (88,1%)	***
MPD (geometric mean)	16	541	483		3	76	66	
Responders to gSG6-P1 peptide								
Prevalence	55/97 (56,70%)	75/83 (90,36%)	85/93 (91,40%)	***	7/14 (50%)	17/18 (94,44%)	26/27 (96,30%)	***
MPD (geometric mean)	17	549	489		5	69	65	

*MPD* mean parasite density

P-value: χ^2^ test, *** very significant

### IgG level against gSG6-P1 peptide according to the use of insecticide-treated nets

The use of ITNs by children in the urban malaria context (low exposure to *Anopheles* bites but high risk of malaria transmission) could have a major impact on the level of human–vector contact. Indeed, the results showed that, when the different neighbourhoods were considered, the proportion of individuals who had declared that they slept under an ITN significantly varied between the neighbourhoods studied in the rainy season (Chi square test: X-squared = 24, 98; df = 2; *p* < 0.0001) but not in the dry season (Table 2). Therefore, the use of ITNs could be one of the major factors of variation of specific IgG levels between the different neighbourhoods. Specific IgG responses were compared between children depending on whether or not they slept (the night before sampling) under an ITN. This comparison was done in the whole population and in the two age groups (≤ 5 and > 5 years old). Surprisingly, the results indicated that the anti-gSG6-P1 IgG levels were similar in ITN users compared to non-ITN users in the rainy (*p* = 0.337 Mann–Whitney) and dry seasons (*p* = 0.094 Mann–Whitney) (Additional file 2). No significant results were observed in the two age groups during the two seasons between children depending on whether or not they declared they slept under an ITN (children ≤ 5: *p* = 0.376 in the rainy season; *p* = 0.435 in the dry season, and children > 5: *p* = 0.569 in the rainy season; *p* = 0.93 in the dry season). When comparing the age groups ≤ 5 versus > 5 years old between the two seasons, the results showed that there was no difference in specific IgG levels, regardless of the season (*p* = 0.407 in the rainy and *p* = 0.288 the dry season).

**Table 2:** Comparison of the “declared or not” use of insecticide-treated nets (ITN) between neighbourhoods according to seasons

Insecticide-treated Net (ITN)								
Rainy season					Dry season			
Net use	Dar-es-salam	Kennedy	N’gattakro	p-value	Dar-es-salam	Kennedy	N’gattakro	p-value
Yes	8 (8.88%)	32 (42.1%)	21 (23.59%)	***	26 (55.31%)	27 (48.21%)	38 (64.40%)	NS
No	82 (91.12%)	44 (57.9%)	68 (76.41%)		21 (44.69%)	29 (51.79%)	21 (35.6%)	

*NS* not significant

P-value: χ^2^ test, *** p < 0.0001

## Discussion

To improve malaria surveillance and control in an urban context, it is essential to evaluate the risk of transmission between neighbourhoods in the same urban area. The results of the present study indicated that human exposure to *Anopheles* bites varied between the three neighbourhoods studied depending on the season. In the rainy season, the level of specific IgG was higher than in the dry season, but it did not vary according to the neighbourhoods. In contrast, the specific IgG level varied significantly according to the neighbourhoods in the dry season. It was higher in NGA compared to KEN and DAR. The semi-urban environment of Bouaké is particular in its urban agricultural practices (rice farming and gardening), the presence of shallows, different habitat types and different population densities between neighbourhoods. Household drinking water storage practices and discharge of sewage into the streets could also create potential breeding sites of *Anopheles*, with an intensity that can vary according to the neighbourhood. This could explain the difference observed in human exposure to malaria vectors between neighbourhoods and highlights the necessity of assessing the differences of exposure to *Anopheles* bites in this context.

In the rainy season, several new temporary breeding sites of *Anopheles* (puddles, ponds, etc.) arise with rainwater. These add to the number of permanent breeding sites that already exist in neighbourhoods. Therefore, *Anopheles* densities increase and individuals receive more bites, which would explain the higher levels of specific IgG response observed in populations in the rainy season compared to the dry season. A recent study has shown that the populations of the city of Bouaké are highly exposed to *Anopheles* bites, similar to populations of rural areas, probably linked to the semi-urban context of Bouaké city [21]. The present study confirms these results and shows high specific IgG responses in Bouake’s population during the rainy season, regardless of the neighbourhood studied. In a recently urbanized area in Kenya, a study showed a strong preference of *An. gambiae* for man-made, aquatic sites such as permanent habitats in the rainy season [25]. Individuals living in the KEN neighbourhood appear to be slightly more exposed to *Anopheles* bites compared to the DAR and NGA neighbourhoods, even if this difference is not significant. This could be explained by the fact that in KEN, rice farming and vegetable gardening are more prevalent in the centre of the neighbourhood in contrast to DAR and NGA where they are located at the periphery.

Interestingly, in the dry season, even though the median of the specific IgG level sharply decreased compared to the rainy season, it was observed that the specific IgG levels were only significantly different between neighbourhoods in the dry season. Many factors could explain this heterogeneity of human exposure in an urban area. In the dry season, individuals in NGA, and especially children under 5 years old, were more exposed to *Anopheles* bites and were at higher risk of malaria transmission than those of DAR and KEN. This could be explained by the fact that most children in this age group usually still sleep with their mother. Indeed, it has been shown in Kenya that the greater exposure to malaria vector bites occurs indoors in the early evening when LLINs (long-lasting insecticide nets) are not used [26]. Thus, children staying indoors with their parents a long time before sleeping under the nets could be exposed to *Anopheles* bites. This early biting habit of some malaria vectors could then reduce the protective efficacy of LLINs. In addition, the decrease of mosquito bites received by individuals and the heat during the dry season could encourage some people to stay outdoors longer before going to bed. All these phenomena in the urban environment could explain the differences in the risk of malaria transmission between neighbourhoods during this hot period. However, the present results show that the frequency of ITN use (sleeping under ITNs) did not differ between neighbourhoods in the dry season, suggesting that ITN use is similar between neighbourhoods in this particular season. This difference in exposure to *Anopheles* bites between the individuals in each neighbourhood could be explained by an inadequate ITN use by the populations living in NGA or use of damaged ITNs (presence of holes). In addition, it could be due to the fact that individuals go to sleep late under ITNs because of the heat or for other sociological reasons in this neighbourhood. In areas where individuals typically stay outside later in the evenings, the protective effect of using an ITN or protection inside houses is nullified if they are exposed during the evening hours outside the home [4].

Interestingly, a trend according to the density of human population was observed. DAR’s specific IgG response appeared the lowest in both the rainy and dry seasons (highly evident in responders to the gSG6-P1 peptide) and was significantly lower compared to KEN and NGA. This observation could be explained by a kind of dilution effect of *Anopheles* bites when population size increases. For example, individuals living in KEN could be exposed more to *Anopheles* bites because of the low human density [4], in contrast to DAR presenting a high population density. Several factors related to rapid and uncontrolled population and/or household growth can have major implications for the disease transmission patterns in sub-Saharan African cities [4, 27], such as Bouaké.

The high level of specific IgG response observed between neighbourhoods in the rainy season seemed to be in accordance with the parasitological results. High *Plasmodium* prevalence in the populations of the three neighbourhoods was observed in the rainy season and remained stable during the dry season. The decrease of the specific IgG level and parasite density in the dry season compared to the rainy season was related to a considerable decrease in the level of exposure to *Anopheles* bites. This could be explained by the disappearance of the temporary breeding sites and the decrease of *Anopheles* density [21, 28]. The very high prevalence of *P. falciparum* in the neighbourhood populations studied during the two seasons can be explained by the presence of permanent sites (shallows, rice crops, vegetable gardens) during the dry season, which continue to serve as breeding sites for *Anopheles.* Khaemba et al. [25] showed that dams and swamps remained the preferred sites of *An. gambiae* during the dry season.

The present study is a first step to explore the neighbourhood-dependent heterogeneity of human exposure to the *Anopheles* vector in an urban area. It cannot be conclude which pertinent socioepidemiological and/or environmental factors explain the differences of human exposure to *Anopheles* bites between the neighbourhoods, specifically observed in the dry season. Multiple factors may be implicated and future studies will be necessary to precisely indicate the environmental and/or sociological factors involved, some of which may be specific to urban contexts, responsible for this heterogeneity of *Anopheles* exposure between neighbourhoods in the same urban area. In addition, it could be interesting to conduct similar studies in different African cities presenting various population sizes (megalopolis *versus* medium cities, for example) and different degrees and histories of urbanization.

## Conclusion

The use of the *An. gambiae* salivary biomarker (gSG6-P1) showed that the human exposure to malaria vectors was very low in the dry season compared to the rainy season, although malaria prevalence remained stable during both seasons. In the rainy season, the level of exposure to malaria vectors was high, but did not vary according to neighbourhoods. Interestingly, it varied according to neighbourhoods in the dry season. The salivary biomarker of human exposure to *Anopheles* bites could be a relevant tool to orient malaria control strategies aiming to target malaria control in urban neighbourhoods at high risk of malaria.

## Additional files

**Additional file 1.** IgG level with gSG6-P1 salivary peptide according to age. Fig A and B: IgG levels to gSG6-P1 peptide of children under 5 years according districts (Fig. A rainy season, Fig. B dry season). Fig. C and D: IgG levels to gSG6-P1 peptide of children over 5 years according districts (Fig. C rainy season, Fig. D dry season).

**Additional file 2.** IgG level with gSG6-P1 salivary peptide according to the use of insecticide-treated nets (ITN) in the whole population (A) and in age groups (B and C). Fig. A: IgG level to gSG6-P1 peptide in the whole population according to the ITN use in the rainy (Fig. A1) and dry (Fig. 1B) seasons. Fig. B: IgG level to gSG6-P1 peptide in “under 5 years” age group according to the ITN use in the rainy (Fig. A1) and dry (Fig. 1B) seasons. Fig. C: IgG level to gSG6-P1 peptide in “over 5 years” age group according to the ITN use in the rainy (Fig. A1) and dry (Fig. 1B) seasons.

## Abbreviations

gSG6-P1: *An. gambiae* Salivary Gland Protein-6 peptide 1
IgG: immunoglobulin G
ELISAs: Enzyme-Linked Immunosorbent Assay
ΔOD: delta optical density
ITN: insecticide-treated net
NGA: N’gattakro
KEN: Kennedy
DAR: Dar-es-salam
