# Supplementary material for: Exploring the heterogeneity of human exposure to malaria vectors in an urban setting, Bouaké, Côte d’Ivoire, using an immuno-epidemiological biomarker
Source: Malar J. 2019 Mar 11;18:68. doi: 10.1186/s12936-019-2696-z (PMC6413440; doi:10.1186/s12936-019-2696-z)
Supplement: Supplementary file 1 — Additional file 1. IgG level with gSG6-P1 salivary peptide according to age. Fig A and B: IgG levels to gSG6-P1 peptide of children under 5 years according districts (Fig. A rainy season, Fig. B dry season). Fig. C and D: IgG levels to gSG6-P1 peptide of children over 5 years according districts (Fig. C rainy season, Fig. D dry season). [file 12936_2019_2696_MOESM1_ESM.pdf]

Additional file 1

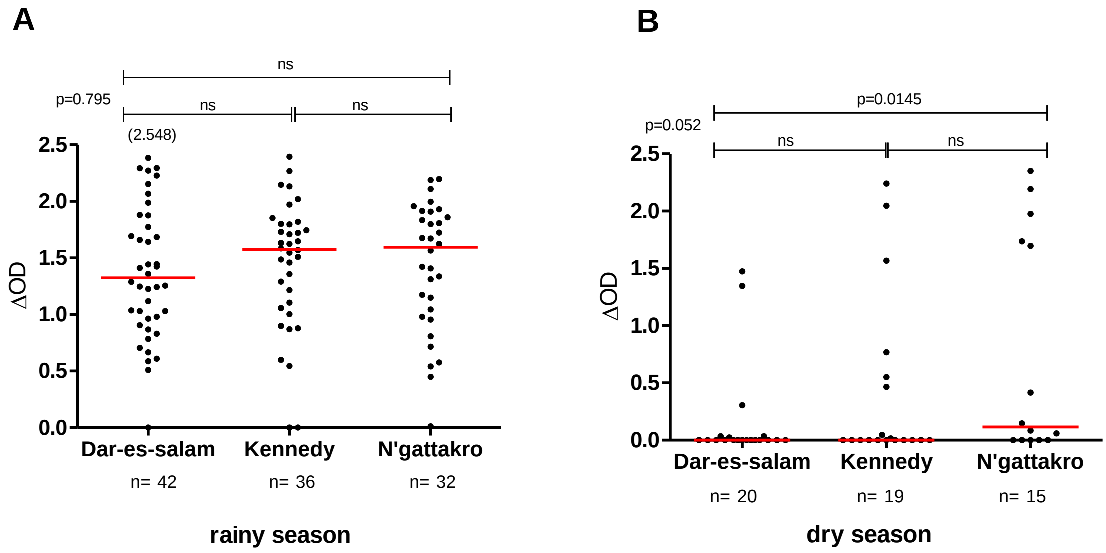

IgG levels to gSG6-P1 peptide of children under 5 years according districts.

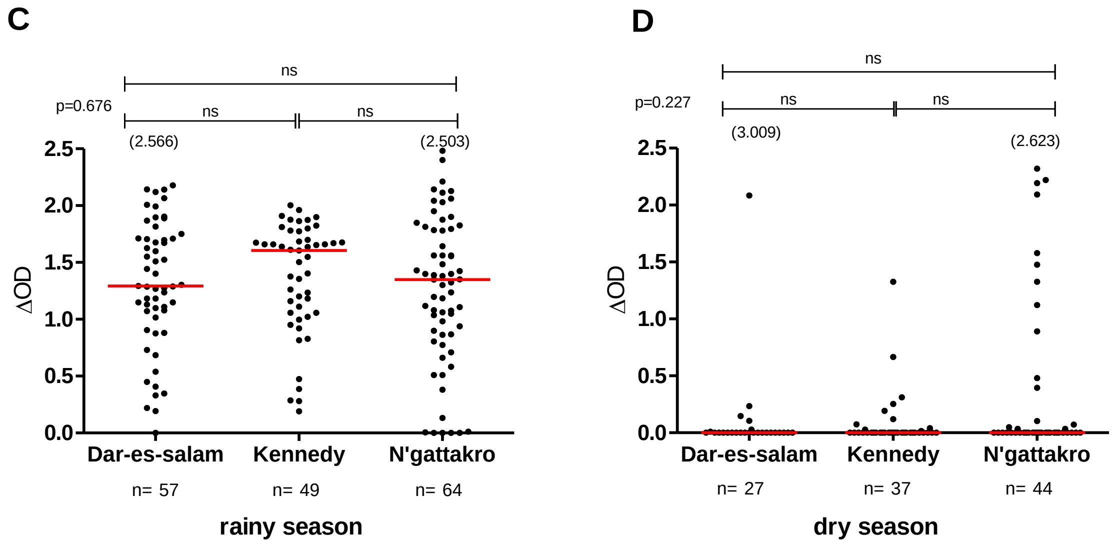

IgG levels to gSG6-P1 peptide of children over 5 years according districts.
