# Supplementary material for: Exploring the heterogeneity of human exposure to malaria vectors in an urban setting, Bouaké, Côte d’Ivoire, using an immuno-epidemiological biomarker
Source: Malar J. 2019 Mar 11;18:68. doi: 10.1186/s12936-019-2696-z (PMC6413440; doi:10.1186/s12936-019-2696-z)
Supplement: Supplementary file 2 — Additional file 2. IgG level with gSG6-P1 salivary peptide according to the use of insecticide-treated nets (ITN) in the whole population (A) and in age groups (B and C). Fig. A: IgG level to gSG6-P1 peptide in the whole population according to the ITN use in the rainy (Fig. A1) and dry (Fig. 1B) seasons. Fig. B: IgG level to gSG6-P1 peptide in “under 5 years” age group according to the ITN use in the rainy (Fig. A1) and dry (Fig. 1B) seasons. Fig. C: IgG level to gSG6-P1 peptide in “over 5 years” age group according to the ITN use in the rainy (Fig. A1) and dry (Fig. 1B) seasons. [file 12936_2019_2696_MOESM2_ESM.pdf]

Additional file 2

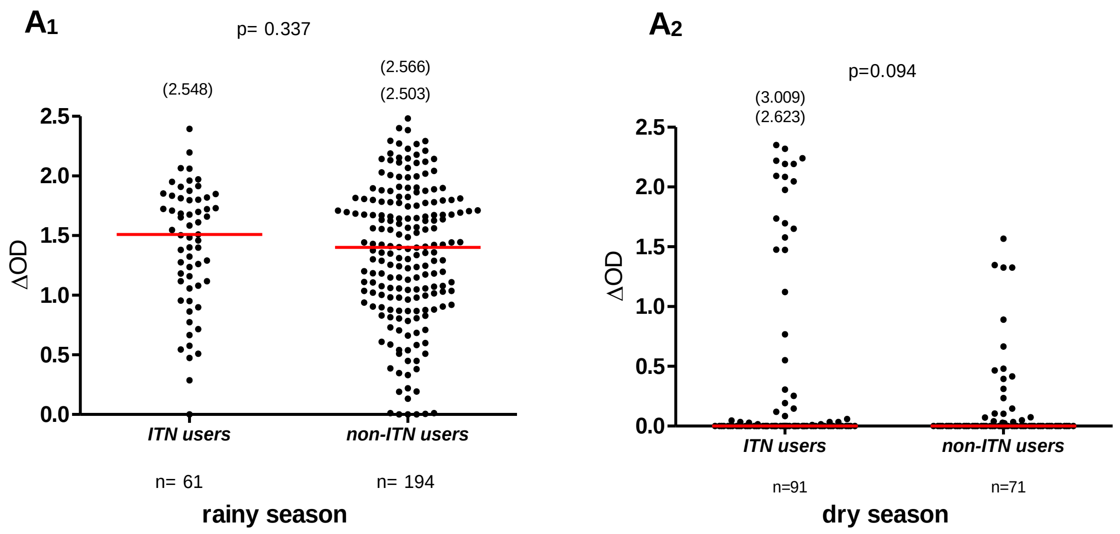

Figure A: ***IgG level to gSG6-P1 peptide in the whole population according to the use of insecticide-treated nets (ITN)***

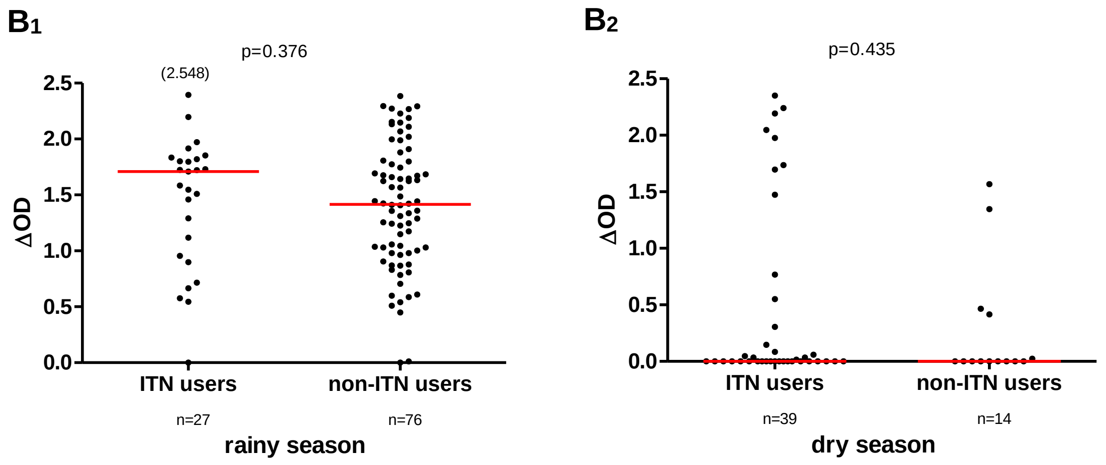

Figure B: ***IgG level to gSG6-P1 peptide to children under 5 years according to the use of insecticide-treated nets (ITN)***

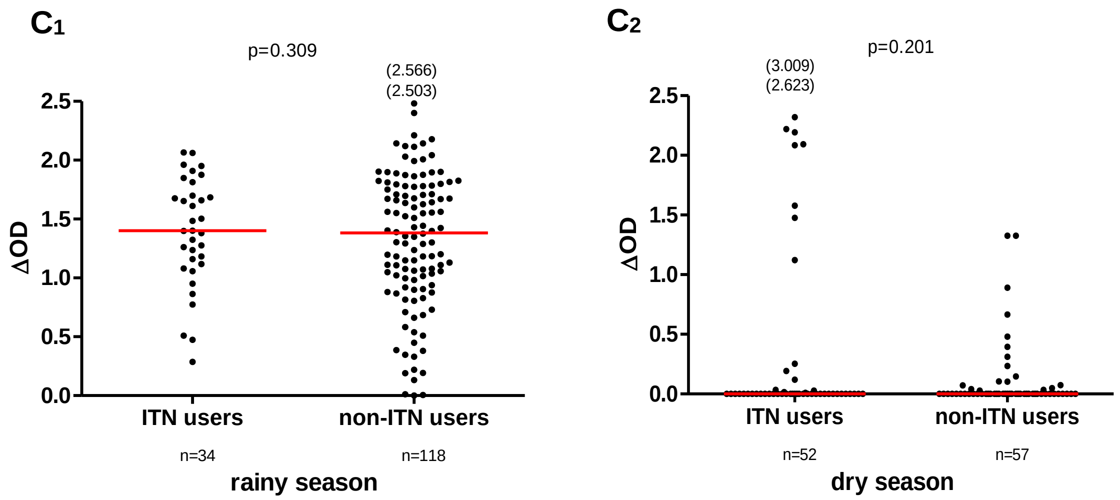

Figure C: ***IgG level to gSG6-P1 peptide to children over 5 years according to the use of insecticide-treated nets (ITN)***
